# Association of a novel nutritional index with stroke in Chinese population with hypertension: Insight from the China H-type hypertension registry study

**DOI:** 10.3389/fnut.2023.997180

**Published:** 2023-04-11

**Authors:** Yumeng Shi, Xingjiu Wang, Chao Yu, Wei Zhou, Tao Wang, Lingjuan Zhu, Huihui Bao, Xiaoshu Cheng

**Affiliations:** ^1^Department of Cardiovascular Medicine, The Second Affiliated Hospital of Nanchang University, Nanchang, Jiangxi, China; ^2^Jiangxi Provincial Cardiovascular Disease Clinical Medical Research Center, Nanchang, Jiangxi, China; ^3^Wuyuan Ziyang County Health Center, Shangrao, Jiangxi, China; ^4^Center for Prevention and Treatment of Cardiovascular Diseases, The Second Affiliated Hospital of Nanchang University, Nanchang, Jiangxi, China

**Keywords:** nutritional index, malnutrition, stroke, hypertension, older

## Abstract

**Objective:**

The abbreviated TCB index (TCBI) is a novel indicator of nutritional status, calculated by multiplying the serum values of triglycerides (TG), total serum cholesterol (TC), and body weight. The research on the relationship between this index and stroke is limited. We aimed to investigate the association between TCBI and stroke in Chinese hypertensive patients.

**Methods and results:**

A total of 13,358 adults with hypertension from the China H-type Hypertension Registry Study were enrolled. The TCBI was calculated as TG (mg/dL) * TC (mg/dL) * body weight (kg)/1,000. The primary outcome was an incidence of stroke. Multivariable adjusted models revealed an inverse association between TCBI and the prevalence of stroke. In the fully adjusted model, the results showed that the prevalence of stroke decreased by 13% (OR, 0.87; 95% CI, 0.78–0.98, *p* = 0.018) per SD increment of LgTCBI. When TCBI was assessed as a categorical variable, compared with those in group Q4 (TCBI ≥ 2,399), the participants in group Q3 (TCBI ≥ 1,476 and <2,399), group Q2 (TCBI ≥ 920 and <1,476), and group Q1 (TCBI < 920) had increases in stroke prevalence of 42% (OR, 1.42; 95% CI, 1.13–1.80; *p*-value = 0.003), 38% (OR, 1.38; 95% CI, 1.07–1.80; *p*-value = 0.014), and 68% (OR, 1.68; 95% CI, 1.24–2.27; *p*-value = 0.001), respectively. Subgroup analysis showed an interaction between age and TCBI and stroke (age < 60 years OR, 0.69; 95% CI, 0.58–0.83; vs. age ≥ 60 years OR, 0.95; 95% CI, 0.84–1.07; *P* for interaction = 0.001).

**Conclusion:**

We found an independent negative association between TCBI and the prevalence of stroke, especially in hypertensive patients aged < 60 years.

## Introduction

Stroke is a significant public health problem characterized by high morbidity, high disability, high mortality, and high recurrence rate (1). The latest research on global disease burden (GBD), published in 2019, reported 12.2 million new cases of stroke and 100 million stroke patients worldwide (2). The study also pointed out that 6.55 million people worldwide died from stroke, accounting for 11.6% of deaths (2). Further, 2.4 million new stroke cases and 1.1 million stroke-related deaths are reported in China every year (3). Stroke has become the leading cause of death in China (4), and the burden of stroke in China is gradually increasing. Therefore, to prevent the occurrence and development of stroke and related cardiovascular death, we need to build a greater understanding of the changeable risk factors and corresponding biological indicators of stroke.

Stroke is a chronic inflammatory process with multiple causes, including hypertension, dyslipidemia, and nutritional status (5). Nutritional status is closely related to cardiovascular disease (CVD). Accumulating evidence has suggested that malnutrition can lead to CVDs, such as heart failure (6), coronary heart disease (CHD) (7, 8), and peripheral artery disease (PAD) (9, 10). However, previous tools for evaluating the nutritional status, such as the Geriatric Nutritional Risk Index (GNRI) (11), Controlling Nutritional Status (CONUT) (12), and Prognostic Nutritional Index (PNI) (13), have not been widely used in cardiovascular clinical practice because of their complicated calculation methods. Based on the above background, doi and his colleagues (14) put forward a new, straightforward index to indicate nutritional status, the index is calculated with the blood lipid index and body weight, which can be easily obtained in clinical practice. The TCB index (TCBI) is derived from the serum values of triglycerides (TG) and total serum cholesterol (TC) abbreviations, as evident in the following formula: TCB index = TG (mg/dL) × TC (mg/dL) × body weight (kg)/1,000. Previous studies have shown that TCBI is a prognostic indicator for patients with heart failure (15), CHD (14), critically ill patients (16), and the general population (17). Some studies have shown that malnutrition can be used as an independent prognostic factor for stroke patients, but there are few studies regarding nutritional status and stroke. Cardiovascular disease is heterogeneous, and the nutritional indices corresponding to different diseases should be inconsistent. The nutritional indices in general population should be in the normal range, but whether a low level in the normal range can be used as a risk factor for stroke deserves further investigation. This exploration is of great significance for the comprehensive management of the occurrence and development of stroke.

Therefore, in order to fill the gaps in the above research, we systematically assessed the association between TCBI and stroke in Chinese hypertensive patients for the first time by using the data of the China H-type Hypertension Registry Study, and we further evaluated the possible effect modifiers on the relationship between TCBI and stroke.

## Materials and methods

### Study design and population

Data were based on the China H-type Hypertension Registry Study (CHHRS). The CHHRS is an ongoing observational real-world study initiated in March 2018 in Wuyuan, China. More details of this study have been published elsewhere (18, 19). Inclusion criteria were as follows: (1) patients with hypertension aged >18 years; (2) hypertension, defined as systolic blood pressure (SBP) of ≥140 mmHg or diastolic BP (DBP) of ≥ 90 mmHg, at screening visits; and (3) use of antihypertensive medication. Exclusion criteria were as follows: (1) psychological or nervous system impairment resulting in an inability to provide informed consent and (2) inability to follow up according to the study protocol or plans to relocate in the near future. After all, patients were provided with information regarding the study and provided written informed consent, the study commenced. Blood samples and outcome events were collected at baseline through a triennial follow-up. All procedures involving human subjects/patients were approved by the ethics committees of the Institute of Biomedicine, Anhui Medical University, and the Second Affiliated Hospital of Nanchang University.

A total of 14,234 patients with hypertension satisfied the inclusion and exclusion criteria. After excluding subjects with atrial fibrillation (AF) (*n* = 388), which can occasionally cause stroke (3), those with missing information on TCBI (*n* = 4), and those using lipid-lowering medications (*n* = 484), the final analysis included 13,358 patients (Supplementary Figure 1).

### Data collection

According to a standard protocol, through face-to-face interviews, trained investigators used a validated questionnaire to obtain the baseline data, including demographic factors, anthropometric indices (height, weight, waist circumference, and BP), lifestyle behaviors (smoking and alcohol consumption), and medical history. Current smoking was defined as smoking ≥1 cigarette per day for ≥1 year or a cumulative smoking amount of ≥360 cigarettes per year. Alcohol consumption was defined as drinking an average of ≥2 times per week over 1 year. Body mass index (BMI) was defined as body weight divided by height 2 (kg/m^2^).

After overnight fasting, blood was collected *via* venipuncture from all patients. Serum creatinine, homocysteine (Hcy), serum uric acid (SUA), serum albumin, TG, TC, high-density lipoprotein cholesterol (HDL-C), and fasting plasma glucose (FPG) was measured using automatic clinical analyzers (Beckman Coulter Canada, Inc., Mississauga, ON, Canada) at the Biaojia Biotechnology in Shenzhen, Guangdong Province, China. Estimated glomerular filtration rate (eGFR) was calculated using the Chronic Kidney Disease Epidemiology Collaboration (CKD-EPI) equation (20).

### Definition of the TCBI and stroke

As reported by previous studies, TCBI can be used as a new nutritional index to evaluate nutritional status (14, 16, 17). TCBI is calculated as follows: TCBI = TG (mg/dL) * TC (mg/dL) * body weight (kg)/1,000 (16).

The main outcome measure was incidence of stroke (ischemic or hemorrhagic), excluding strokes caused by transient ischemic attacks (TIA), craniocerebral trauma, and intracranial tumors (21). Stroke, in this study, was a self-reported stroke recorded by questionnaire. The specific questions were as follows: occurrence of stroke; time of stroke; symptoms at that time; kind of treatment received; and presence of relevant medical records, including discharge summaries and imaging data. In addition, the stroke events collected through the questionnaire were further confirmed through the local health insurance registration system and inpatient medical records.

### Other definitions

Atrial fibrillation was defined as atrial fibrillation or atrial flutter recorded by standard 12-lead electrocardiograms acquired on-site or by previous patients’ atrial fibrillation onset histories (22). Diabetes was defined as a self-reported physician diagnosis, FPG concentration of ≥7.0 mmol/L, or the use of glucose-lowering drugs. The medical history of coronary heart disease (CHD) was self-reported and was mainly collected using a questionnaire. Each participant was asked about the presence or absence of CHD when CHD occurred, symptoms, the kind of treatment administered, and the presence of relevant medical records, including discharge summaries and imaging data. In addition, the CHD events collected through the questionnaire were further confirmed through the local health insurance registration system and inpatient medical records.

### Statistical analyses

Continuous variables are presented as means ± standard deviations (SDs), and categorical variables are expressed as percentages. Quantile-quantile (Q-Q) plot and the Anderson–Darling test (23) were used to test the normality of the evaluation variables in this study. Specifically, the method to test for normal distribution involved comparing the histogram of the sample data with the standard normal curve or comparing the normalized quantile of the sample data with the standard quantile of the normal distribution, using a Q-Q diagram. In the Q-Q diagram, the correlation between the sample data and the normal data could reflect whether the data conformed to a normal distribution. For the normal data, the scattered points in the Q-Q diagram approximated to a straight line, indicating a high positive correlation. Because the sample size of this study was ≥200, we used the Anderson–Darling test to determine whether the variables in this study conformed to a normal distribution. When *p* ≥ 0.05, the data were considered to be normally distributed. The quartiles of TCBI groups were compared with Student’s *t*-test and the chi-square test for categorical variables. One-way analysis of variance for normally distributed data and the chi-square test were used to calculate the differences among the quartiles of baseline TCBI for continuous and categorical variables.

Because the TCBI had skewed distributions, it was transformed into normal distributions by logarithm (log)-transformation. We constructed three models and used multivariate logistic regression analysis to evaluate the relationship between TCBI and stroke. The models were developed as follows: Model 1, crude model; and sex; Model 2, adjusted for age, sex, BMI, SBP, DBP, current smoking, current drinking, diabetes, antihypertensive drugs, antidiabetes agents; and Model 3, adjusted for age, sex, BMI, SBP, DBP, current smoking, current drinking, diabetes, antihypertensive drugs, antidiabetes agents, antiplatelet drug, Hcy, FPG, HDL-C, low-density lipoprotein cholesterol (LDL-C), SUA, and eGFR. The covariates to be adjusted in the regression model are selected based on the ≥10% independent change in the estimated effect (24). Because of the baseline imbalance in the prevalence of stroke between the two groups, we matched the 1:3 propensity score (PSM) with a caliper value of 0.02. In addition, we performed sensitivity analyses to assess the relationship between TCBI and stroke, in matched data. We explored the shape of the curve or linear relationship between TCBI and stroke using a generalized additive model and a fitted smoothing curve (penalized spline method). The likelihood ratio test compares models with and without interactions. Further stratified analyses by subgroups, including sex (male or female), age (<60 vs. ≥60 years), BMI (<24 vs. ≥24 kg/m^2^), current smoking (no vs. yes), SBP (<140 vs. ≥140 mmHg), DBP (<90 vs. ≥90 mmHg), diabetes (no vs. yes), and coronary heart disease (no vs. yes), were also explored by multivariable logistic regression models to test for consistency of results.

A two-sided *p*-value of <0.05 was considered statistically significant. The statistical package R^1^ and Empower (R) (X&Y Solutions, Inc., Boston, MA, USA)^2^ were used for all data analyses.

## Results

### Baseline characteristics

The flow chart of the participants is presented in Supplementary Figure 1. A total of 13,358 participants were included in the final analysis.

The baseline demographic and clinical characteristics are detailed in Table 1. Among all the participants, 47.28% were men, and the mean (SD) age was 63.64 (9.35) years. Among them, 784 (5.87%) participants suffered from stroke, 2,366 (17.71%) participants suffered from diabetes, and 569 (4.26%) participants suffered from CHD. In summary, there were significant differences in baseline characteristics between quartiles of TCBI, with the exception of SBP, CHD, and antiplatelet drug (all *p* > 0.05). Those in group Q1 were more likely to be males, to be older, to be current drinkers and smokers, to have lower rates of antihypertensive agents and antidiabetes agents, to have a lower proportion of diabetes, to have higher Hcy levels and HDL-C, and to have lower BMI, DBP, FPG, serum albumin, TC, TG, LDL-C, eGFR, and SUA levels than the three groups with higher TCBI values.

**TABLE 1:** Baseline characteristics of study participants by quartiles of baseline TCBI.

Variable^a^	TCBI quartiles				*p*-value
	Q1 (<920)	Q2 (≥920–<1,476)	Q3 (≥1,476–<2,399)	Q4 (≥2,399)	
*N*	3,338	3,337	3,338	3,338	
Age (year)	67.11 ± 9.21	64.53 ± 9.04	62.65 ± 8.88	60.24 ± 8.90	<0.001
Male [*n* (%)]	1,937 (58.03%)	1,495 (44.80%)	1,415 (42.39%)	1,464 (43.86%)	<0.001
Current smoker [*n* (%)]	1,150 (34.45%)	835 (25.04%)	741 (22.20%)	763 (22.86%)	<0.001
Current drinker [*n* (%)]	862 (25.83%)	704 (21.11%)	672 (20.13%)	713 (21.37%)	<0.001
BMI (kg/m^2^)	21.14 ± 3.83	23.04 ± 3.15	24.34 ± 3.04	25.83 ± 3.23	<0.001
SBP (mmHg)	147.97 ± 18.78	148.69 ± 17.36	148.67 ± 17.28	149.00 ± 17.78	0.114
DBP (mmHg)	86.38 ± 11.00	88.43 ± 10.49	89.53 ± 10.23	91.76 ± 10.48	<0.001
Hcy (μmol/L)	18.34 ± 11.45	17.86 ± 10.46	17.56 ± 10.74	17.86 ± 11.19	0.036
FPG (mmol/L)	5.70 ± 1.06	6.00 ± 1.34	6.25 ± 1.53	6.73 ± 2.09	<0.001
Serum albumin (g/L)	44.80 ± 4.13	46.59 ± 3.70	47.14 ± 3.66	48.07 ± 3.96	<0.001
TC (mmol/L)	4.43 ± 0.85	5.06 ± 0.89	5.40 ± 0.95	5.86 ± 1.15	<0.001
TG (mmol/L)	0.87 ± 0.23	1.28 ± 0.28	1.79 ± 0.44	3.27 ± 1.62	<0.001
HDL-C (mmol/L)	1.68 ± 0.46	1.63 ± 0.43	1.54 ± 0.41	1.44 ± 0.37	<0.001
LDL-C (mmol/L)	2.38 ± 0.56	2.90 ± 0.59	3.21 ± 0.68	3.53 ± 0.85	<0.001
eGFR (mL/min/1.73 m^2^)	86.68 ± 20.74	88.51 ± 19.81	89.32 ± 19.85	89.97 ± 19.93	<0.001
SUA (μmol/L)	393.39 ± 113.36	400.86 ± 115.39	419.35 ± 115.62	459.54 ± 126.43	<0.001
CHD	153 (4.58%)	152 (4.55%)	129 (3.86%)	135 (4.04%)	0.358
Diabetes^$^	291 (8.72%)	459 (13.75%)	638 (19.11%)	977 (29.27%)	<0.001
Antihypertensive agents [*n* (%)]	2,041 (61.14%)	2,122 (63.63%)	2,164 (64.83%)	2,198 (65.87%)	<0.001
Antidiabetes agents [*n* (%)]	92 (2.76%)	125 (3.75%)	179 (5.36%)	244 (7.31%)	<0.001
Antiplatelet drug [*n* (%)]	64 (1.92%)	75 (2.25%)	66 (1.98%)	63 (1.89%)	0.711

BMI, body mass index; SBP, systolic blood pressure; DBP, diastolic blood pressure; Hcy, homocysteine; FPG, fasting plasma glucose; TC, total cholesterol; TG, triglycerides; HDL, high-density lipoprotein cholesterol; LDL-C, low-density lipoprotein cholesterol; SUA, serum uric acid; eGFR, estimated glomerular filtration rate; CHD, coronary heart disease.

^a^Data are presented as number (%) or mean ± standard deviation.

^$^Diabetes was defined as self-reported physician diagnosis of diabetes or FBG concentration ≥7.0 mmol/L or use of glucose-lowering drugs.

### Association of nutritional index with stroke

**FIGURE 1:**
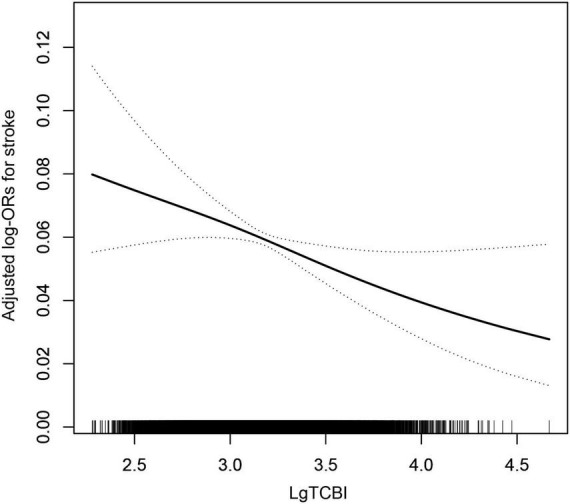
The association between TCBI and the prevalence of stroke. The solid line and dashed line represent the estimated values and their corresponding 95% confidence interval, respectively. The adjustment factors included age, sex, BMI, SBP, DBP, current smoking, current drinking, diabetes, antihypertensive drugs, antidiabetes agents, antiplatelet drug, Hcy, FPG, HDL-C, LDL-C, SUA, and eGFR.

According to the nutritional index, calculated with the grouped categorical variables, as previously mentioned, we performed the multivariate logistic regression models. The odds ratios (ORs) and 95% confidence intervals (Cis) for stroke in the three models are listed in Table 2. In Model 1, the prevalence of stroke decreased by 12% (OR, 0.88; 95% CI, 0.82–0.95; *p* = 0.001) per SD increment of LgTCBI. After further adjustment for age, sex, BMI, SBP, DBP, current smoking, current drinking, diabetes, antihypertensive drugs, and antidiabetes agents, the results showed that the prevalence of stroke decreased by 9% (OR, 0.91; 95% CI, 0.83–0.99; *p* = 0.033) per SD increment of LgTCBI. In the fully adjusted Model 3, the results showed that the negative association between TCBI and stroke remained stable (OR, 0.87; 95% CI, 0.78–0.98; *p* = 0.018). When TCBI was assessed as a categorical variable, compared with those in group Q4 (TCBI ≥ 2,399), the participants in group Q3 (TCBI ≥ 1,476 and <2,399), group Q2 (TCBI ≥ 920 and <1,476), and group Q1 (TCBI < 920) had increases in stroke prevalence of 42% (OR, 1.42; 95% CI, 1.13–1.80; *p* = 0.003), 38% (OR, 1.38; 95% CI, 1.07–1.80; *p* = 0.014), and 68% (OR, 1.68; 95% CI, 1.24–2.27; *p* = 0.001), respectively. Smooth curve fitting (penalized spline method) was performed to explore the dose-response association of TCBI with stroke and found that the incidence of stroke gradually increases with decreases in TCBI level (Figure 1). After we performed further additional analysis without excluding patients taking lipid lowering drugs, we adjusted the administration of lipid lowering drugs in the fully adjusted model as shown in Supplementary Table 1, and the results showed that the negative correlation between TCBI and stroke remained stable.

**TABLE 2:** Association of triglycerides, total cholesterol, and body weight index (TCBI) with stroke.

TCBI^†^	Events (%)	OR for prevalent stroke, *P*-value		
		Model 1	Model 2	Model 3
Per SD increment	784 (5.87%)	0.88 (0.82, 0.95) 0.001	0.91 (0.83, 0.99) 0.033	0.87 (0.78, 0.98) 0.018
**Quartiles**				
Q1 (<920)	229 (6.86%)	1.53 (1.24, 1.89) <0.0001	1.43 (1.12, 1.83) 0.004	1.68 (1.24, 2.27) 0.001
Q2 (≥920–<1,476)	195 (5.84%)	1.29 (1.04, 1.61) 0.021	1.29 (1.02, 1.63) 0.031	1.38 (1.07, 1.80) 0.014
Q3 (≥1,476–<2,399)	207 (6.20%)	1.38 (1.11, 1.71) 0.004	1.38 (1.10, 1.72) 0.005	1.42 (1.13, 1.80) 0.003
Q4 (≥2,399)	153 (4.58%)	1.00	1.00	1.00
*P* for trend		<0.001	0.015	0.003

^†^TCBI value was log10-transformed. Model 1: crude model. Model 2: adjusted for age, sex, BMI, SBP, DBP, current smoking, current drinking, diabetes, antihypertensive drugs, and antidiabetes agents. Model 3: adjusted for age, sex, BMI, SBP, DBP, current smoking, current drinking, diabetes, antihypertensive drugs, antidiabetes agents, antiplatelet drug, Hcy, FPG, HDL-C, LDL-C, SUA, and eGFR.

### Subgroup analyses by potential effect modifiers

**FIGURE 2:**
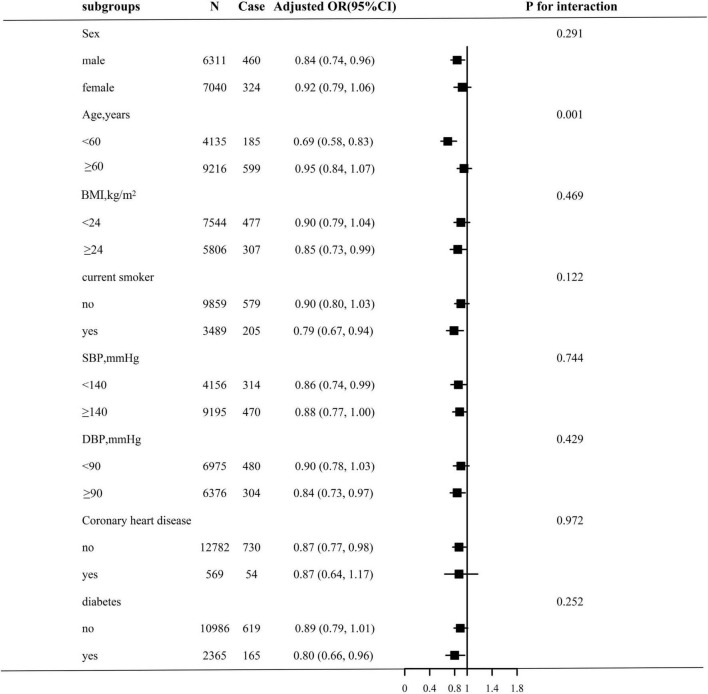
Stratified Analyses by potential modifiers of the association between TCBI and the prevalence of stroke. Each subgroup analysis adjusted for age, sex, BMI, SBP, DBP, current smoking, current drinking, diabetes, antihypertensive drugs, antidiabetes agents, antiplatelet drug, Hcy, FPG, HDL-C, LDL-C, SUA, eGFR, and except for the stratifying variable.

Stratified analyses were performed to assess the relation of TCBI (per SD increment) with stroke in various subgroups (Figure 2). None of the variables, including sex, BMI, current smoking, SBP, DBP, coronary heart disease, and diabetes, significantly modified the association between TCBI and stroke prevalence (all *P*-interaction > 0.05), with the exception of age. Significant group differences were observed among participants with different ages (<60 years: OR, 0.69; 95% CI, 0.58–0.83; vs. ≥60 years: OR, 0.95; 95% CI, 0.84–1.07; *P* for interaction = 0.001).

### Sensitivity analysis

We performed a 1: 3 match of the data *via* PSM, and the data changes before and after the match are shown in Table 3 before the match, we found that there were significant differences in baseline characteristics between the participants with stroke and non-stroke, with the exception of BMI, smoking, FPG, SUA (all *p* > 0.05). The stroke groups were more likely to be males; to be older; to be non-current drinkers, had higher rates of antihypertensive agents, antiplatelet drug and antidiabetes agents, with a higher proportion of diabetes and CHD, higher Hcy levels, and lower SBP, DBP, serum albumin, TC, TG, HDL-C, LDL-C, eGFR levels than participants without stroke. After matching the data, we found that the drinking states of SBP, DBP, serum albumin, TG, TC, HDL-C, eGFR, antihypertensive agents and antiplatelet drug had significant differences between patients with stroke and non-stroke. We further use that data to carry out multivariate logistic regression analysis (Table 4). The results showed that the prevalence of stroke decreased by 28.9% (OR, 0.711; 95% CI, 0.541–0.936; *p* = 0.05) per SD increment of LgTCBI. When TCBI was assessed as a categorical variable, compared with those in group Q4 (TCBI ≥ 2,399), the participants in group Q3 (TCBI ≥ 1,476 and <2,399), group Q2 (TCBI ≥ 920 and <1,476), and group Q1 (TCBI < 920) had increases in stroke prevalence of 31.8% (OR, 1.318; 95% CI, 1.03–1.687; *p* = 0.028), 28.3% (OR, 1.283; 95% CI, 0.994–1.655; *p* = 0.050), and 45.4% (OR, 1.454; 95% CI, 1.140–1.853; *p* = 0.003), respectively. So the negative correlation between TCBI and stroke remained stable.

**TABLE 3:** Baseline characteristics of study participants with and without stroke before and after matching (after a 1: 3 match).

	Before matching			After matching			
Variable^a^	Non-stroke	Stroke	*P*-value	Non-stroke	Stroke	Standardized diff.	*P*-value
	(*n* = 12,574)	(*n* = 784)		(*n* = 2,181)	(*n* = 727)		
Age (year)	63.55 ± 9.40	65.01 ± 8.50	<0.001	64.24 ± 9.38	64.99 ± 8.44	0.084	0.057
Male [*n* (%)]	5,856 (46.57%)	460 (58.67%)	<0.001	995 (45.6)	307 (42.2)	0.068	0.121
Current smoker [*n* (%)]	3,287 (26.15%)	205 (26.15%)	0.999	631 (28.9)	186 (25.6)	0.075	0.091
Current drinker [*n* (%)]	2,863 (22.78%)	91 (11.62%)	<0.001	555 (25.4)	91 (12.5)	0.334	<0.0001
BMI (kg/m^2^)	23.60 ± 3.56	23.36 ± 6.07	0.076	23.53 ± 3.67	23.35 ± 6.24	0.036	0.338
SBP (mmHg)	148.77 ± 17.72	145.61 ± 18.94	<0.001	148.05 ± 18.13	145.77 ± 18.93	0.123	0.004
DBP (mmHg)	89.15 ± 10.73	87.08 ± 10.60	<0.001	88.86 ± 11.24	87.20 ± 10.64	0.152	0.001
Hcy (μmol/L)	17.75 ± 10.79	20.38 ± 13.30	<0.001	19.35 ± 13.94	20.28 ± 13.38	0.068	0.119
FPG (mmol/L)	6.17 ± 1.60	6.12 ± 1.56	0.406	6.19 ± 1.81	6.11 ± 1.54	0.046	0.307
Serum albumin (g/L)	46.69 ± 4.04	45.99 ± 4.11	<0.001	46.38 ± 4.13	46.02 ± 4.12	0.088	0.041
TC (mmol/L)	5.20 ± 1.10	5.03 ± 1.16	<0.001	5.18 ± 1.14	5.04 ± 1.17	0.121	0.005
TG (mmol/L)	1.81 ± 1.26	1.69 ± 1.08	0.008	1.81 ± 1.38	1.69 ± 1.09	0.098	0.031
HDL-C (mmol/L)	1.58 ± 0.43	1.48 ± 0.40	<0.001	1.58 ± 0.47	1.49 ± 0.40	0.204	<0.0001
LDL-C (mmol/L)	3.01 ± 0.80	2.94 ± 0.85	0.013	2.99 ± 0.81	2.94 ± 0.85	0.066	0.121
eGFR (mL/min/1.73 m^2^)	88.95 ± 20.02	83.37 ± 21.01	<0.001	85.91 ± 22.21	83.61 ± 20.84	0.107	0.014
SUA (μmol/L)	418.00 ± 120.65	422.75 ± 118.94	0.285	433.21 ± 126.34	423.38 ± 120.59	0.080	0.066
CHD	515 (4.10%)	54 (6.89%)	<0.001	106 (4.9)	44 (6.1)	0.053	0.245
Diabetes^$^	2,201 (17.50%)	165 (21.05%)	0.012	415 (19)	147 (20.2)	0.030	0.515
Antihypertensive agents [*n* (%)]	7,892 (62.78%)	637 (81.25%)	<0.001	1,414 (64.8)	585 (80.5)	0.356	<0.0001
Antidiabetes agents [*n* (%)]	573 (4.56%)	68 (8.67%)	<0.001	124 (5.7)	55 (7.6)	0.076	0.082
Antiplatelet drug [*n* (%)]	171 (1.36%)	97 (12.37%)	<0.001	79 (3.6)	43 (5.9)	0.108	0.010

BMI, body mass index; SBP, systolic blood pressure; DBP, diastolic blood pressure; Hcy, homocysteine; FPG, fasting plasma glucose; TC, total cholesterol; TG, triglycerides; HDL, high-density lipoprotein cholesterol; LDL-C, low-density lipoprotein cholesterol; SUA, serum uric acid; eGFR, estimated glomerular filtration rate; CHD, coronary heart disease.

^a^Data are presented as number (%) or mean ± standard deviation.

^$^Diabetes was defined as self-reported physician diagnosis of diabetes or FBG concentration ≥7.0 mmol/L or use of glucose-lowering drugs.

**TABLE 4:** Association of triglycerides, total cholesterol, and body weight index (TCBI) with stroke (after a 1: 3 match).

TCBI	OR for prevalent stroke, *P*-value
Per SD increment	0.711 (0.541–0.936) *p* = 0.05
**Quartiles**	
Q1 (<921)	1.454 (1.140–1.853) *p* = 0.003
Q2 (≥921–<1,476)	1.283 (0.994–1.655) *p* = 0.05
Q3 (≥1,476–<2,399)	1.318 (1.03–1.687) *p* = 0.028
Q4 (≥2,399)	1
*p* for trend	0.024

Adjusted for none.

## Discussion

In this large-scale, hypertensive population-based cross-sectional study, we found that malnutrition was associated with the risk of stroke. We determined that a lower TCBI level was related to an increased prevalence of stroke, especially in hypertensive patients aged <60 years. Furthermore, relationships remained statistically significant after adjusting for potential confounding variables.

Previous studies have shown that TCBI is a prognostic indicator for patients with heart failure (15), CHD (14), critically ill patients (16), and the general population (17). At the same time, some studies have shown that malnutrition can be used as an independent prognostic factor for stroke patients, but there are few studies on nutritional status and stroke. For example, Yuan et al. (25) assessed the relationship between malnutrition and long-term mortality in 1,065 stroke patients aged ≥65 years. The results showed that malnutrition was prevalent in elderly stroke patients in China and was related to increased mortality. Han et al. (26) conducted a retrospective cohort study of 991 patients with acute ischemic stroke, which showed that malnutrition of adult ischemic stroke patients at admission was associated with a greater risk of acute ischemic stroke and major cardiovascular events. The aforementioned definition of malnutrition is assessed according to the CONUT, GNRI, and PNI. These three scoring indexes are not widely used in cardiovascular clinical practice because of their complicated calculation methods and complex parameters. In order to better evaluate the nutritional status of patients with CVD in clinical practice, the TCBI (14) is used, which can be more easily obtained only by blood lipid and weight. Studies have shown that TCBI, as an evaluation index of malnutrition, is a potent risk predictor of long-term death and adverse cardiovascular events, both in patients with CVD and in the general population (14–17). However, the relationship between this index and stroke has not yet been explored. Therefore, our study offered an opportunity to evaluate the dose-response relation of TCBI with stroke in hypertensive patients by leveraging the well-established CHHRS. The current research makes up for the gaps in previous studies and provides new insights. First, to the best of our knowledge, this study is the first of its kind to explore the relationship between TCBI and stroke in hypertensive patients. The results showed that TCBI was negatively correlated with stroke in hypertensive patients; risk of stroke increased significantly as TCBI level decreased. Second, we also found that age can significantly modify the relationship between TCBI and stroke, and the negative correlation between TCBI and stroke is more significant in patients aged <60 years. It is well known that age is closely related to malnutrition, and with increasing age, the prevalence of malnutrition will increase significantly (27, 28). Moreover, there are more cardiovascular risk factors among elderly patients aged >60 years than in participants aged <60 years (29). Even if the nutritional status of the two groups is the same, the risk of stroke is still significantly higher.

The underlying mechanism of malnutrition increasing the risk of stroke is unclear, but it is biologically reasonable. According to the related literature, malnutrition can induce inflammation and oxidative stress (30, 31). Inflammation plays an essential role in the pathogenesis of stroke. Data from experimental studies indicate that inflammation can increase the level of leukocytes and other related inflammatory cells *in vivo*, by which neutrophils can adhere to activated endothelial cells through chemokines, interact with platelets, promote the release of neutrophil extracellular traps (NET) (32), induce endothelial cell death, adhere to and damage blood vessel walls, damage physiological anticoagulants, cause inflammatory environment, and lead to activation of the coagulation cascade, thrombosis, and growth (33, 34). The thrombus, generated by the above pathological reaction, blocks the blood vessels to brain, causing the occurrence of stroke. Brain cells are vulnerable to the harmful effects of oxidative damage because the neuronal membrane is rich in polyunsaturated fatty acids, which is very easily oxidized; needs a large amount of oxygen to produce energy; and has relatively poor antioxidant defense (35). Ischemic injury causes a rapid, excessive increase in the production and release of free radicals, such as the simultaneous consumption of superoxide anion (ROS) and reactive nitrogen (RNS) and endogenous antioxidant defense, thereby activating the cellular pathways responsible for neuronal necrosis and apoptosis (35–37) leading to neuronal necrosis of brain cells.

## Limitations

This study has several limitations. First, the results are based on data obtained from the cross-sectional study design. We only revealed the relationship between TCBI and stroke, but we could not determine its causal relationship. Second, patients in group Q2 (TCBIQ ≥ 920 and <1,476) had relatively few stroke events, which may have limited our ability to detect differences in stroke groups. Third, the incidence of stroke in this study was determined by questionnaire, but the stroke outcomes we collected were further confirmed by the local health insurance registration system and the patients’ inpatient medical records to reduce the influence of memory bias as much as possible. Fourth, as this study is an postmortem analysis of the China H-type Hypertension Registration Study, the definition of stroke is exclusive to TIA, and the inclusion of different types of stroke is more conducive to the accurate treatment in clinical practice. The population in this study was from rural areas of China and the ratio of stroke to non-stroke was 1: 16, but we used PSM to match the data 1: 3 and further analyzed the sensitivity of the matched data. The result is still stable. However, whether this conclusion can be extrapolated to other ethnic groups remains to be discussed.

## Conclusion

In conclusion, we reported an association between TCBI and stroke in patients with hypertension in rural areas of China and found that a low level of TCBI is related to an increased prevalence of stroke, especially in people aged <60 years.

## Data availability statement

The data that support the findings of this study are available from the corresponding author upon reasonable request. Requests to access these datasets should be directed to XC, xiaoshumenfan126@163.com.

## Ethics statement

The studies involving human participants were reviewed and approved by the Ethics Committees of the Institute of Biomedicine, Anhui Medical University, and The Second Affiliated Hospital of Nanchang University. The patients/participants provided their written informed consent to participate in this study.

## Author contributions

YS participated in the literature search, data analysis, data interpretation, and wrote the manuscript. WZ extracted and collected data. XW, CY, WZ, TW, LZ, and HB conceived of the study and participated in its design and coordination. HB and XC participated in the study design and provided critical revision. All authors read and approved the final manuscript.

## Footnotes

1
http://www.r-project.org

2
www.empowerstats.com
