# Supplementary material for: Association of a novel nutritional index with stroke in Chinese population with hypertension: Insight from the China H-type hypertension registry study
Source: Front Nutr. 2023 Apr 11;10:997180. doi: 10.3389/fnut.2023.997180 (PMC10126229; doi:10.3389/fnut.2023.997180)
Supplement: Supplementary file 1 [file Data_Sheet_1.docx]

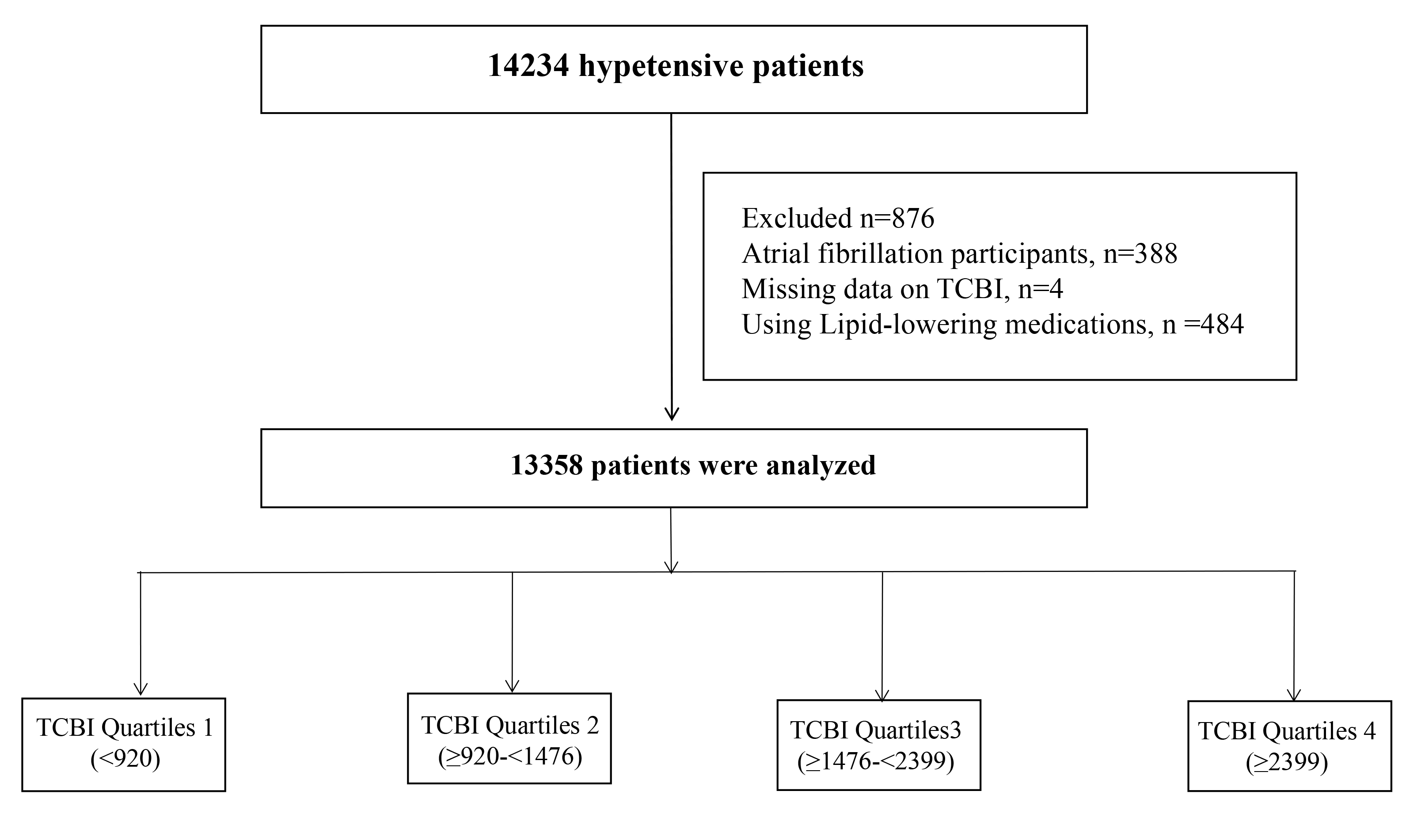


**Figure S1** **The flow chart of study participants**

Table S1 Association of triglycerides, total cholesterol, and body weight index (TCBI) with Stroke (Including patients taking lipid lowering drugs)

| TCBI | Events (%) | OR for Prevalent Stroke, *P* vaule |
| --- | --- | --- |
| Per SD increment | 941(6.8%) | 0.86 (0.77, 0.96) 0.005 |
| Quartiles |  |  |
| Q1 (<920) | 285 (8%) | 1.61 (1.21, 2.14) 0.001 |
| Q2 (≥920-˂1475) | 242 (7%) | 1.40 (1.10, 1.79) 0.007 |
| Q3 (≥1475-˂2401) | 240 (7%) | 1.38 (1.11, 1.73) 0.004 |
| Q4(≥2401) | 174 (5%) | 1 |
| *P for trend* |  | 0.003 |

adjusted for age, sex, BMI, SBP, DBP, current smoking, current drinking, diabetes, antihypertensive drugs, antidiabetes agents, antiplatelet drug, Hcy, FPG, HDL-C, LDL-C, SUA, eGFR, lipid-lowering drug.
